# Clinical characteristics and prognostic factors of brain central neurocytoma

**DOI:** 10.18632/oncotarget.11228

**Published:** 2016-08-11

**Authors:** Yaqi Song, Xinle Kang, Gang Cao, Yongqiang Li, Xilei Zhou, Yusuo Tong, Wanwei Wang

**Affiliations:** ^1^ Department of Radiation Oncology, Huai'an First People's Hospital, Nanjing Medical University, Huai'an, China; ^2^ Department of Ophthalmology, Huai'an Second People's Hospital, Xuzhou Medical College, Huai'an, China; ^3^ Department of Internal Medicine, Huai'an Second People's Hospital, Xuzhou Medical College, Huai'an, China; ^4^ Department of Oncology, HangZhou Hospital of ZheJiang Provincial Corps of Chinese People's Armed Police Force, HangZhou, China

**Keywords:** prognostic factors, central neurocytoma, tumor number, surgery, SEER program

## Abstract

**Background & Aims:**

This study is designed for the clinical characteristics and prognostic factors of central neurocytoma (CN).

**Methods:**

CN patients from 2004 to 2012 were enrolled from the Surveillance Epidemiology and End Results (SEER) data. Clinical characteristics including age, sex, race, tumor size, tumor number, surgery, and radiation therapy were summarized. Univariate and multivariate analysis were performed to explore the prognostic factors of CN.

**Results:**

CN tended to be borderline malignant and single lesion. Compared with other brain tumor (NCN), Patients with CN (CNs) were more likely to be female, young, and non-white race. Surgery was the primary treatment of CN. Univariate and Multivariate analysis indicated tumor number and surgery were both independent prognostic factors of CN (*P* < 0.05). Unifocal CNs had a lower mortality risk than multifocal ones (HR 0.167, 95% CI 0.052-0.537), surgery significantly reduced the death risk of CNs (HR 0.284, 95% CI 0.088-0.921).

**Conclusions:**

CN tend to be borderline malignant, single lesion, operated on. Most CNs are female and younger. single lesion and surgery are the independent positive prognostic factors of CN.

## INTRODUCTION

Central neurocytoma (CN) is a rare central nervous system tumor. It was firstly reported by Hassoun and colleagues in 1982 [1], and was classified as grade II by the World Health Organization in 2000 [2]. Previous studies showed that CN mainly sited in deep mid-line structures near the foramen of Monro, with neurological symptoms of intracranial hypertension, such as headaches and/or visual changes [3]. CN often occurred in young adults [4–6]. Most CN were benign or borderline tumor, and of long-time survival with a gross total removal [7, 8]. However, for the low morbidity, only less than one thousand cases of CN have been reported in the world till today [9]. Thus, detail clinical information of CN is still not very clear, and large sample research of it is very necessary.

The Surveillance, Epidemiology, and End Results (SEER) Program is founded by the National Cancer Institute (NCI) in the United States as an open cancer related database. It records cancer incidence and survival data from population-based cancer registries and covers approximately 28% of the US population. With large information of cancer, it is a very important tool to analyze rare carcinoma.

In view of above, we used SEER data for the analysis of brain central neurocytoma. Purpose to explore the clinical characteristics and prognostic factors of brain central neurocytoma.

## RESULTS

**Figure 1:**
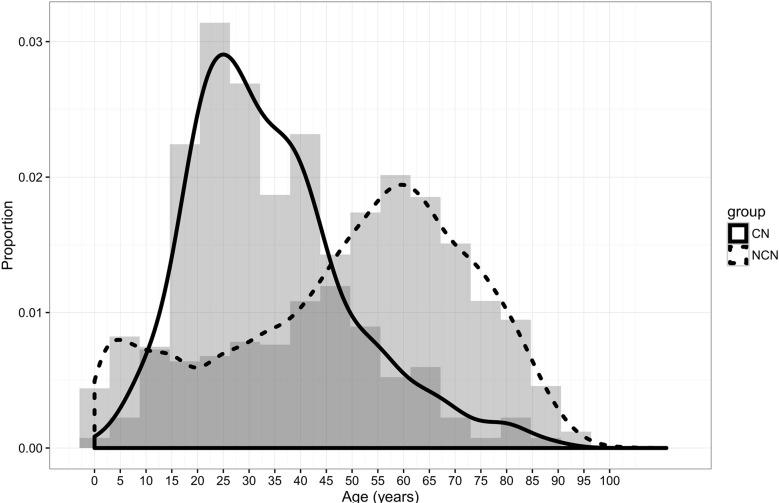
Patients distribution by age in two groups

**Figure 2:**
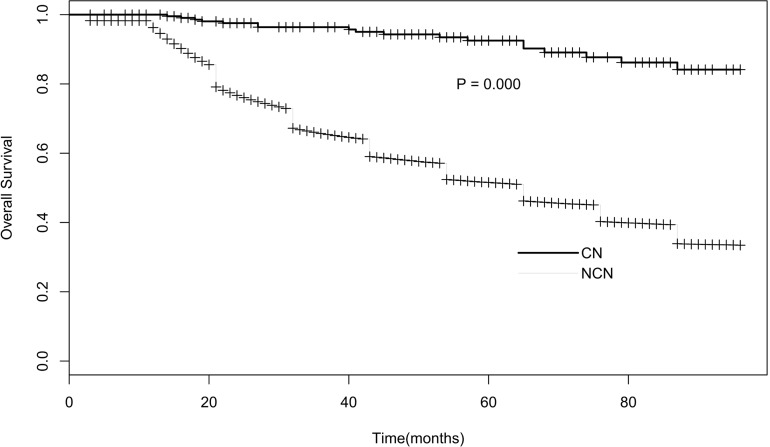
Survival curve of CN *Vs*. NCN

A total of 47,502 primary brain tumor patients were selected from the SEER database. In which, 229 (0.48%) cases were central neurocytoma. Epidemiological data showed that compared with other brain tumor (NCN), patients with central neurocytoma (CNs) tended to be more Female (52.8% *Vs* 44.4%), young (59.8% *Vs* 26.6%, Figure 1), singer lesion (93.9% *Vs* 87.5%), and non-white (21.6% *Vs* 11.4%). All the CNs were benign and borderline malignant (100%), and they were likely to receive more surgery (89.9% vs 71.3%) and less radiotherapy (15.9% *Vs* 55.6%). Median Survival Time (MST) of CNs was more than 96 months, much longer than that of NCNs (65 months) (Figure 2). A detailed listing of the patients' clinical characteristics was presented in Table 1.

**Table 1:** Summary of clinical characteristics between CN and NCN

Variable		CN (%)	NCN (%)	χ^2^	*P*-valueψ
Sex	Female	121 (52.8)	21007 (44.4)	6.177	0.013*
	Male	108 (47.2)	26266 (55.6)		
Race	White	178 (78.4)	49559 (88.6)	11.708	0.001**
	Other	49 (21.6)	6363 (11.4)		
Age	35-	137 (59.8)	12579 (26.6)	126.570	0.000***
	35+	92 (40.2)	34694 (73.4)		
Behavior	Benign	3 (1.3)	3917 (8.3)	2246.800	0.000***
	Borderline	226 (98.7)	4073 (8.6)		
	Malignant	0 (0)	39283 (83.1)		
Size	<=3cm	56 (36.4)	10653 (31.7)	1.323	0.250
	>3cm	98 (63.6)	22938 (68.3)		
Number	single	215 (93.9)	41392 (87.5)	7.821	0.005 **
	multiple	14 (6.1)	5881 (12.4)		
Surgery	None	23 (10.1)	13122 (28.7)	37.238	0.000***
	Surg	204 (89.9)	32593 (71.3)		
radiotherapy	None	191 (84.1)	20092 (44.4)	143.160	0.000***
	RT	36 (15.9)	25211 (55.6)		
MST(months)		96+	65	102.000	0.000***

Abbreviations: CN = central neurocytoma ; NCN = Other brain tumor ; Surg = surgery; RT = radiotherapy; MST: Median Survival Time. ψ chi-square test

**Figure 3:**
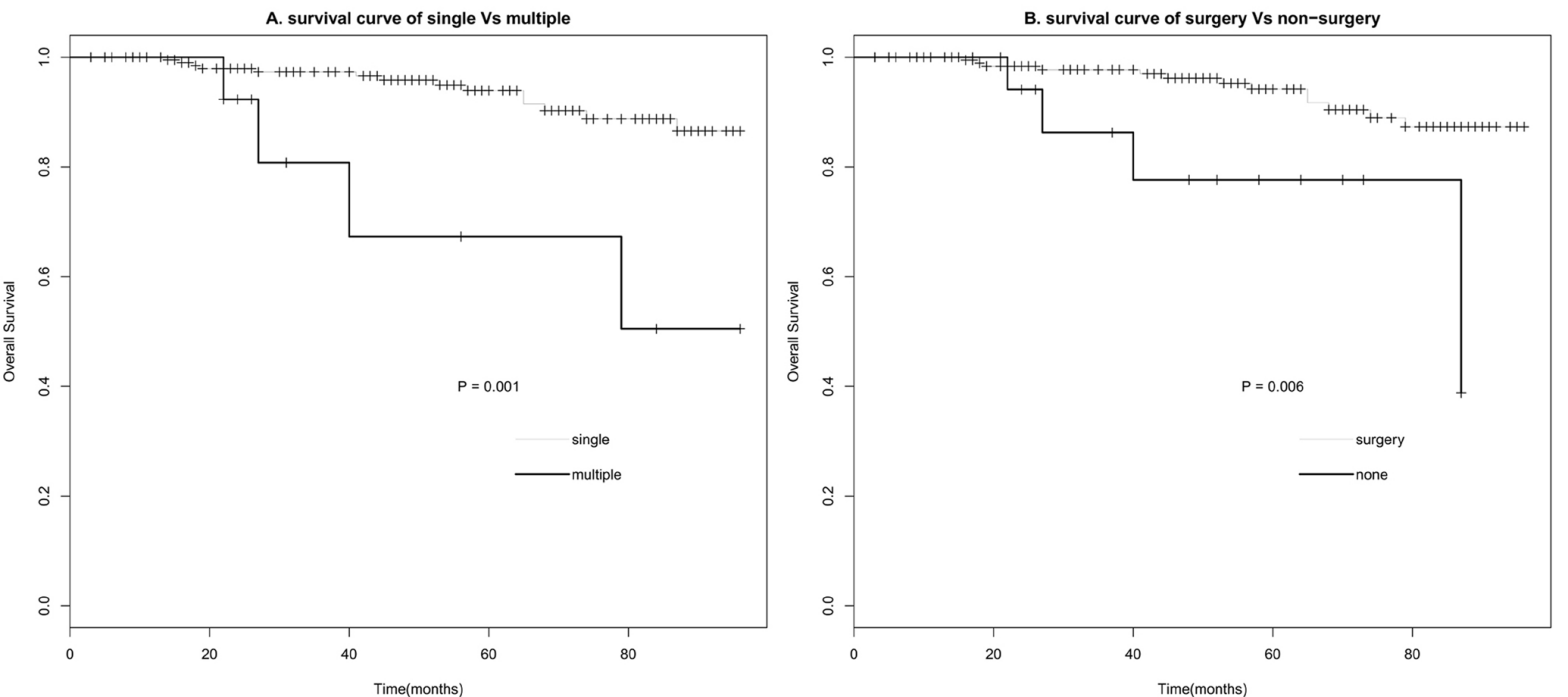
Survival curve of CN based on number (A) and treatment (B)

Univariate survival analysis of clinical characteristics was evaluated with log-rank test (Table 2). Tumor Number (Figure 3A) and Surgery (Figure 3B) were significantly associated with overall survival (*P* < 0.05). Sex, Race, Age, Size, and Radiotherapy showed no significant association with survival (*P* > 0.05). Multivariate analysis of Number and Surgery performed with the Cox regression model indicated Number and Surgery were the independent prognostic factors of survival (*P* < 0.05). Patients with singer tumor had a longer survival time (HR 0.167, 95%CI 0.052- 0.537), Surgery also significantly prolonged patients' survival time (HR 0.284, 95%CI 0.088- 0.921) (Table 3).

**Table 2:** Univariate survival analysis of CN patients

Variable		Univariate analysis	
		**HR(95% CI)**	*****P***-value‡**
**Sex**	Male *Vs* Female	0.709 (0.275-1.830)	0.476
**Race**	White *Vs* Other	2.106 (0.472-8.939)	0.310
**Age**	35- *Vs* 35+	0.459 (0.178- 1.183)	0.101
**Size**	3cm- *Vs* 3cm+	1.547 (0.346- 6.923)	0.565
**Number**	multiple *Vs* single	0.192 (0.063- 0.587)	0.001**
**Surgery**	Surg *Vs* None	0.233 (0.075- 0.719)	0.006**
**Radiation**	RT *Vs* None	1.523 (0.501- 4.630)	0.455

Abbreviations: Surg = surgery; RT = radiotherapy; HR = hazard ratio; CI = confidence interval.

† Log–rank test.

**Table 3:** Multivariate cox proportional hazards regression analysis of CN patients

Variable		Multivariate analysis	
		HR(95% CI)	*P*-value‡
Number	multiple *Vs* single	0.167 (0.052- 0.537)	0.003**
Surgery	Surg *Vs* None	0.284 (0.088- 0.921)	0.036*

Abbreviations: HR = hazard ratio; CI = confidence interval.

‡ Cox regression model test.

## DISCUSSION

The incidence of central neurocytoma is very low, with only 0.1% to 0.5% among all primary brain tumors [4, 10, 11], which makes only several hundred cases being reported since CN was established in 1982 [1, 9]. Although with a low mobility, CN mainly occures in young adults [4–6], performs as a nonmalignant tumor, and may carry favorable prognoses with opportune treatment [7, 8]. All of these make it an important brain tumor worthy of attention. However, as for CN typically site at the deep midline structures near the foramen of Monro, intracranial hypertension is very easy to generate and rapidly aggravate in the early stage, and even lead to herniation and death [8, 10]. Contradiction between the favorable prognoses and rapidly aggressive clinical symptoms made it valuable to clarify the clinical characteristics and prognostic factors of CN, for the optimal treatment strategy and long-time survival.

So far, as we know, almost all the data of CN were from various case reports or small sample retrospective studies, which made the information of CN very unclear and paradoxical. Information from these studies indicated that CN mainly affect young adults at the age between 20 to 40 [4, 7], but no researcher reported the exact peak age. Most studies reported CN seemed to have equally probability in male and female [4, 12], but someone believed male had more chance [13, 14].It usually performed benign, or not malignant [14, 15], but could also exhibit more aggressive clinical behavior, especially in some atypical variants [16–18]. CN typically located in deep midline structures near the foramen of Monro, such as the third ventricle or the anterior portion of the lateral ventricle [3, 8]. but there were also some rare cases of extraventricular neurocytoma reported in other locations, such as spinal dissemination [19–22]. Surgery was the primary initial treatment for CN [23–25], but whether complete resection surgery correlates significantly with overall survival was still disputed [8, 24, 26, 27]. Meanwhile, radiosurgery was also reported by some small sample studies to perform nice effect and little complications to CNs in the past several years [28–31]. Conventional radiotherapy was mainly applied for the management of residual or recurrent CNs [32, 33], but its effect to overall survival was still full of controversial [8, 24, 34–36], and the radiation-induced toxicity was also hard to be ignored for the long-time clinical course [37, 38]. Studies on the prognostic factors of CN was still very few. As far as we known, MIB-1 and surgery were the only two reported possible prognostic indexes of CN [39–41], and there were still some studies opposed to surgery as a prognostic factor [8, 24]. Whether there were other clinical prognostic factors of CN was still unclear. Because of these conflicting views, we designed this study for a further exploration of the characteristics and prognostic factors of CN with SEER database.

In this study, we summarized the clinical characteristics and treatment methods of patients with CN, with the data provided by the SEER database from 2004 to 2012. Characteristics we analysized included sex, race, age, tumor behavior, size, number, surgery and radiation therapy. Firstly, We found CN patients were more likely to be Female, young, single lesion compared with other brain tumors. In our study, the ratio of male *vs* female was 1:1.1, no significant sex variation. This was in line with Yasargil and Sgouros's studies [4, 12], but not agree with Chen and kim's reports [13, 14]. Histogram and density plot of onset age (Figure 1) demonstrated that the peak age of onset was in first half of twenties, with a steep fall after the age of 35 years, though there was another small peak at the age of 40. That was also why we chose 35 years as the critical point of age. This onset age distribution was in according with the previous studies [4, 7], but more detailed. Secondly, as Kim and his partners mentioned [14], we found almost all the CNs were borderline malignant (98.7%), the left were benign, and hence with a much longer median survival time than other brain tumor (96+ months *Vs* 65 months). Patients with CN were likely to receive more surgery (89.9%) and less radiotherapy (15.9%). Besides these, Univariate survival analyses of different clinical factors showed that overall survival (OS) was associated with tumor number and surgery (*P* < 0.05), but of no significantly association with sex, age, race, tumor behavior, size, and radiotherapy (*P* > 0.1). And finally, further multivariate cox proportional hazards regression analysis displayed that tumor number and surgery were both significantly associated with survival (*P* < 0.05). Death risk of patients with single tumor was lower than those with multiple tumors (HR 0.167, 95% CI 0.052- 0.537). Surgery could reduce more than 70% risk of death (HR 0.284, 95% CI 0.088-0.921).

In conclusion, our study demonstrates that CN tend to be female, young, single lesion, borderline malignant, more surgery, and less radiotherapy, compared with other brain tumors. Tumor number and surgery are both independent prognostic factors of CN. Patients with single focus live longer than those with multiple focus. Surgery does significantly prolong the overall survival of CNs.

## MATERIALS AND METHODS

### Patients

SEER data between 1973 and 2012 [“Incidence - SEER 18 Regs Research Data + Hurricane Katrina Impacted Louisiana Cases, Nov 2014 Sub (1973-2012 varying)“] was selected for the study. The National Cancer Institute's SEER*Stat software (Version 8.2.1) was used for the identity of patients. The inclusion criteria contained: (1) primary sited in brain (Site recode ICD-O-3/WHO 2008: 63); (2) with a confirmed diagnosis (Diagnostic Confirmation: Positive histology), (3) central neurocytoma (Histologic Type ICD-O-3: 9506) based on the International Classification of Diseases for Oncology, 3rd Edition (ICD-O-3), (4) being diognosed between 2004 and 2012. And the exclusion criteria contained: (1) unknown age, race; (2) with a surgery status of “Recommended, unknown if performed“. Survival data were extracted at 1-month intervals for a follow-up between 3 months and 96 months.

This study based on public data from the SEER database. The reference number obtained for the permission to access research data files was 10612-Nov2014. No human subjects or personal identifying information were used in this study. No informed consent was require in this study.

### Statistical analysis

The enrolled population was divided into two groups based on different pathology: central neurocytoma (CN group) and not central neurocytoma (NCN group). Chi-square test was used for the difference analysis between the two groups. Univariate analyses with log-rank test and multivariate analysis with cox proportional hazards regression model were performed to explore the clinical prognosis factors of CN, with a statistically significant difference at the value of *p* < 0.05. All analysis were performed with *survival* [42] and *ggplot2* [43] package of R (version 3.2.3) [44].
